# Circulating myomiRNAs as biomarkers in patients with Cushing’s syndrome

**DOI:** 10.1007/s40618-023-02184-3

**Published:** 2023-09-08

**Authors:** C. Pivonello, R. Patalano, C. Simeoli, T. Montò, M. Negri, F. Amatrudo, N. Di Paola, A. Larocca, E. M. Crescenzo, R. Pirchio, D. Solari, C. de Angelis, R. S. Auriemma, L. M. Cavallo, A. Colao, R. Pivonello

**Affiliations:** 1 Department of Public Health, Federico II University, Naples, Italy; 2 Dipartimento di Medicina Clinica e Chirurgia, Sezione di Endocrinologia, Diabetologia, Andrologia e Nutrizione, Università Federico II di Napoli, Naples, Italy; 3 Department of Neuroscience, Division of Neurosurgery, Reproductive and Odontostomatological Sciences, Federico II University, Naples, Italy; 4 UNESCO Chair for Health Education and Sustainable Development, Federico II University, Naples, Italy

**Keywords:** microRNAs, myomiRNAs, miR-133a-3p, miR-200b-3p, Cushing’s disease, Cushing’s syndrome

## Abstract

**Purpose:**

Impairment of skeletal muscle mass and strength affects 40–70% of patients with active Cushing’s syndrome (CS). Glucocorticoid excess sustains muscle atrophy and weakness, while muscle-specific microRNAs (myomiRs) level changes were associated with muscle organization and function perturbation. The aim of the current study is to explore changes in circulating myomiRs in CS patients compared to healthy controls and their involvement in IGFI/PI3K/Akt/mTOR pathway regulation in skeletal muscle.

**Methods:**

C2C12, mouse myocytes, were exposed to hydrocortisone (HC), and atrophy-related gene expression was investigated by RT-qPCR, WB and IF to assess HC-mediated atrophic signalling. miRNAs were evaluated in HC-treated C2C12 by PCR Arrays. MyomiRs significantly overexpressed in C2C12 were investigated in 37 CS patients and 24 healthy controls serum by RT-qPCR. The anti-anabolic role of circulating miRNAs significantly upregulated in CS patients was explored in C2C12 by investigating the IGFI/PI3K/Akt/mTOR pathway regulation.

**Results:**

HC induced higher expression of atrophy-related genes, miR-133a-3p, miR-122-5p and miR-200b-3p in C2C12 compared to untreated cells. Conversely, the anabolic IGFI/PI3K/Akt/mTOR signalling was reduced and this effect was mediated by miR-133a-3p. In CS patients miR-133a-3p and miR-200b-3p revealed higher circulating levels (*p* < 0.0001, respectively) compared to controls. ROC curves for miR-133a-3p (AUC 0.823, *p* < 0.0001) and miR-200b-3p (AUC 0.850, *p* < 0.0001) demonstrated that both myomiRs represent potential biomarkers to discriminate between CS and healthy subjects. Pearson’s correlation analysis revealed that circulating levels of miR-133a-3p are directly correlated with 24 h urinary-free cortisol level (*r* = 0.468, *p* = 0.004) in CS patients.

**Conclusions:**

HC induces atrophic signals by miR-133a-3p overexpression in mouse myocytes and humans. Circulating miR-133a-3p is promising biomarkers of hypercortisolism.

**Supplementary Information:**

The online version contains supplementary material available at 10.1007/s40618-023-02184-3.

## Introduction

Cushing’s syndrome (CS) is a rare endocrine disorder caused by chronic exposure to the excessive endogenous cortisol released by the adrenal glands, or by the prolonged exposure to exogenous synthetic glucocorticoids (GCs) for medical treatment [1]. CS can be classified in ACTH-dependent and ACTH-independent forms; the ACTH-dependent forms are due to the presence of an ACTH-secreting pituitary tumour (70% of cases), named Cushing’s disease (CD), or an ACTH-secreting or corticotropin-releasing hormone-secreting ectopic tumour (10% of cases), named ectopic CS, whereas the ACTH-independent forms are due to the presence of a cortisol-secreting adrenal lesion, including bilateral adrenal hyperplasia or dysplasia, adenoma or carcinoma (20% of cases), named adrenal CS [1, 2]. CS is characterized by increased mortality and impaired quality of life due to the associated burdensome metabolic, cardiovascular, psychiatric, skeletal, as well as muscular comorbidities [2–5]. Myopathy secondary to cortisol excess, mainly affecting the proximal musculature of the lower limbs, is a well-known complication of CS with muscle weakness, representing the main symptom and muscle wasting the main sign of myopathy [6]. Interestingly, while conflicting data reported not affected [7], decreased [8] or increased muscle mass [9] after hypercortisolism biochemical remission, muscle performance remained impaired even after the short-term and long-term achievement of hypercortisolism remission [7, 9].

The balance between anabolism and catabolism regulates the maintenance of skeletal muscle mass and performance [10]. In anabolic conditions, muscle growth is under the control of several pathways mainly including the PI3K/Akt/mTOR pathway [11]. The anabolic factors insulin and insulin-like growth factor I (IGF-I) induce the upstream activation of PI3K/Akt/mTOR pathway sustaining the muscle growth by stimulating protein synthesis through the activation of p70S6K protein. Concomitantly, the activation of PI3K/Akt/mTOR pathway promotes protein accumulation by suppressing protein degradation through the blocking of specific transcription factors, including the forkhead box protein O (FoxO) proteins. The blocking of FoxO proteins negatively controls the transcription of the E3 ubiquitin ligases muscle RING finger 1 (MuRF1) and muscle atrophy F-box (MAFbx/Atrogin-1), known as atrogenes, which mediate the muscle protein polyubiquitination and degradation via the ubiquitin–proteasome system (UPS), and by inhibiting the transcription of the autophagy-related gene which mediates the muscle protein degradation via autophagy-lysosome system [12, 13]. If the muscle protein synthesis rate exceeds protein degradation, muscle mass will hypertrophy over time. In catabolic conditions, the protein degradation rate exceeds the protein synthesis, resulting in reduced skeletal muscle mass.

The suppressed or reduced activation of anabolic signalling pathways, including PI3K/Akt/mTOR, leads to decreased protein synthesis. Concomitantly, the activation of the atrogenes and autophagy-related gene transcription trigger the two major protein degradation pathways, the UPS and the autophagy-lysosome systems [12, 13].

As reported in rodents and humans, in pathological conditions GCs excess sustain muscle weakness and muscle wasting affecting type II muscle fibers, expressing abundant glucocorticoid (GC) receptors (GRs) [14–17]. GCs concomitantly affect muscle mass and muscle performance. GCs reduce protein synthesis antagonizing the anabolic signalling pathways, and stimulate protein degradation, mainly inducing MuRF1 and Atrogin-1 genes transcription, leading to a rapid loss of muscle mass. Concomitantly, catabolic GCs effects impair muscle performance by damaging muscle contractile function through the reduction of mitochondria and sarcoplasmic reticulum number and sarcoplasm via the autophagy-lysosome system. The chronic catabolic effects culminate in the muscle atrophy characterized, at the histological level, by the shrinkage of myofibers due to net protein breakdown and by mitochondrial aggregation and vacuolization and increased interstitial tissue between fibers [12–19].

Mature microRNAs (miRNAs), belonging to the large family of non-coding RNAs, are important players in the post-transcriptional control of gene expression. In this last decade, there is an increasing interest in using extracellular miRNAs as circulating biomarkers of several diseases. Importantly, many miRNAs are ubiquitously expressed in most tissues and cell types, but some are tissue specific. Subsets of miRNAs, including miR-1, miR-133a, miR-133b, miR-206, miR-208a, miR-208b, miR-486 and miR-499, can be described as muscle-specific or muscle-enriched, therefore, they are defined myomiRs [20, 21]. Among them, miR-1/133a and miR-206/133b clusters are highly conserved in the musculatures of flies, mice and humans [22]. MyomiRs are involved in myoblast proliferation, differentiation and regeneration [21]. MyomiRs have been found at low levels in most biofluids, including blood. Alterations in myomiRs levels have been reported in several pathologic conditions associated with muscle organization and function perturbations [23–25]. Although more recently two studies investigated changes in circulating miRNA levels in CS conditions [26, 27], no findings have focused on the alteration in myomiRs levels.

The current study aims to explore changes in circulating myomiRs in patients with CS compared to healthy controls and their involvement in insulin-like growth factor 1 receptor (IGF-IR) signalling in skeletal muscle in the condition of GC excess.

For this purpose, the study was structured in two in vitro sections performed on myocytes, and one ex vivo section performed on human serum. In the first in vitro study section, C2C12 mouse myocytes were exposed to hydrocortisone (HC), and mRNA and protein expression of the atrogenes was investigated to confirm the activation of HC-mediated atrophic signalling. The subset of myomiRs significantly overexpressed in HC-treated C2C12 cells compared to the untreated cells was investigated in the ex vivo study section as circulating myomiRs in patients affected by CS and in healthy controls. Finally, in the second in vitro study section, the potential regulatory role of specific myomiRs, which were found to be significantly upregulated in HC-treated C2C12 cells and in CS patients, on IGF-I/PI3K/Akt/mTOR signalling pathway-dependent muscle wasting was evaluated.

## Materials and methods

### Drugs

HC was provided by Sigma Aldrich, dissolved in ethanol 100% and stored at -80 °C as a stock solution of 10^–3^ M. Fresh serial dilutions were prepared prior to each experiment by diluting the stock solution in DMEM serum-free. A concentration of 1.4*10^–6^ M of HC, resembling a high cortisol level (500 ng/ml), was used, although in in vitro study, the physiological contribution of the GC binding protein cannot be considered. Relacorilant (Rel), a highly selective GR modulator that competitively antagonizes cortisol activity, was provided by Selleckchem and dissolved in DMSO 100% and stored at − 80 °C as a stock solution of 10^–3^ M. Fresh serial dilutions were prepared prior to each experiment by diluting the stock solution in DMSO 10%. A final concentration of 1*10^–6^ M of Rel was used as reported by the previous study [28].

### Cell line: culture and transfection

C2C12 myoblast cell line was provided by American Type Culture Collection (ATCC) and cultured in DMEM medium (Gibco, Thermo Fisher) supplemented with 10% of fetal bovine serum (FBS), 1*10^5^ U/L penicillin and streptomycin (Gibco, Thermo Fisher) and 0.2% of Amphotericin B (Gibco, Thermo Fisher). To induce skeletal muscle differentiation and obtain terminally differentiated myocytes, the growth medium was replaced by a differentiation medium as previously described [29]. The cell line was grown in the humidified condition at 37 °C and 5% CO_2_.

C2C12 myocytes were transfected by transfection reagent HilyMax reconstituted by Lipoform Buffer (#357 Dojindo EU), or by reagent HilyMax and miScript miRNA Inhibitor at the final concentration of 30 nM (Anti-mmu-miR-133a-3p; Qiagen, USA) reconstituted by RNase-free water following the supplier’s instructions.

### RNA isolation and RT-qPCR

RNA isolation and RT-qPCR were performed as previously reported [29]. Briefly, C2C12 cells were plated in 60 mm dishes in a complete culture medium and incubated at 37 °C in a humidified 5% CO_2_ atmosphere (day 0). After 48 h of adhesion, on day 2, DMEM full medium was replaced by differentiation medium. After 48 h, on day 4, differentiated C2C12 were treated with a single dose of HC for 2, 4, 6 and 12 h. Total RNA was extracted using TRIzol reagent protocol (Life Technologies, California, United States). RT-qPCR was performed to quantify the mRNA expression levels of the atrophy-related genes Atrogin-1 (Fw: CCTGCATGTGCTCAGTGAGGA; Rv: CTTCTTGGGTAACATCGTACAAGC) and MuRF-1 Fw: CTTCTTGGGTAACATCGTACAAGC; Rv: CTTCGTGTTCCTTGCACATC), and the housekeeping gene cyclophilin (Fw: CGCCACTGTCGCTTTTCG; Rv: AACTTTGTCTGCAAACAGCTC. The total reaction volume (12 μl) consisted of 2 μl of cDNA and 10 μl of SYBR Green PCR Mastermix (Applied Biosystems, Branchburg, NJ, USA). After two initial heating steps at 50 °C (2 min.) and 95 °C (10 min.), samples were subjected to 40 cycles of denaturation at 95 °C (15 s) and annealing at 60 °C 60 s. All samples were assayed in duplicate. Data were normalized against the expression of the housekeeping gene cyclophilin.

### Protein extraction and western blot

Protein extraction and WB were performed, as previously reported [30]. Briefly, C2C12 cells were seeded at 5*10^5^ cell density into 100 mm culture dishes in a complete medium (day 0). After 48 h of adhesion, on day 2, DMEM full medium was withdrawn with differentiation medium. After 48 h, on day 4, differentiated C2C12 were treated with a single dose of HC for 12 h or with a single dose of Rel for 1 h before the HC treatment, and then proteins extraction and WB for atrogenes have been performed, while in differentiated C2C12 transfected with miRNA inhibitor and treated with HC proteins extraction and WB for IGF-IR have been performed. 40 μg of proteins were loaded, respectively, into polyacrylamide gel. Primary antibodies used for WB analysis were the following: Atrogin-1 (12,866-AP Proteintech, Chicago USA,) dilution used 1:500; MuRF-1 (E-AB-60840 Elabscience, Houston, Texas,) dilution used 1:500, IGF-IR (#3027, Cell Signalling), dilution used 1:1000, pAKT (Ser472) (#9271, Cell signaling) dilution used 1:1000, AKT (#9272, Cell signaling) dilution used 1:1000, pp70S6K (Thr389) (#9206, Cell signaling) dilution used 1:500, p70S6K (sc-8418, Santa Cruz) dilution used 1:1000, p4eBP1 (Thr70) (#13,396, Cell signaling) dilution used 1:1000, 4eBP1 (#9644, Cell signaling) dilution used 1:1000, β-actin (A4700, Sigma Aldrich) dilution used 1:10,000; whereas anti-mouse and anti-rabbit HRP-conjugated secondary antibodies (ImmunoReagents, Inc.) dilution used 1:2000 in 2.5% non-fat dry milk in PBS 1X were used for the detection of proteins. Primary antibodies specific for Atrogin-1, MuRF-1 and IGF-IR and β-actin were probed on nitrocellulose filters overnight. Peroxidise-conjugated secondary antibodies used were probed on nitrocellulose filters for 1 h and half. Immunoreactive bands after chemiluminescent reaction by ECL system (Immobilon Western, Millipore, WBKLS0500) were detected using ImageQuant Las 4000 (GE Healthcare).

### Immunofluorescence

Immunofluorescence was performed as previously reported [31] with a small change in the protocol due to the different antibodies used. Briefly, C2C12 cells were plated in 35 mm dishes (Ibidì, 80,136) in a complete medium (day 0). After 48 h of adhesion, on day 2, DMEM full medium was withdrawn with a differentiation medium. After 48 h, on day 4, differentiated C2C12, as well as differentiated C2C12 transfected with miRNA inhibitor, were treated with a single dose of HC for 12 h. The cells were incubated with primary antibodies against Atrogin-1 (12,866-AP Proteintech, Chicago USA,) dilution used 1:50; MuRF-1 (E-AB-60840 Elabscience, Houston, Texas,) dilution used 1:1000, and IGF-IR (sc271606, Santa Cruz Biotechnology Inc.,) dilution used 1:250 in blocking buffer for 2 h at RT. Cells were washed thrice in 0.1% Triton/PBS 1X and incubated 1 h with TRIC- or FITC-conjugated secondary antibodies (ImmunoReagents, Inc., Raleigh, NC) diluted 1:500 in blocking buffer; while to detect the nuclei a 4.6-Diamidino-2-phenylindole (DAPI) (Lonza Group Ltd, Basel, Switzerland) staining, diluted in PBS 1X 1:60,000, was used. Images were visualized on an inverted microscope Olympus IX51 equipped for fluorescence and phase-contrast microscopy (Olympus, Milan, Italy) and were captured at 40X and 60X magnification and acquired with Olympus Digital Camera F-View II (Olympus, Milan, Italy).

### miRNAs isolation and profiling in C2C12 cell line

miRNAs were isolated from differentiated C2C12 cell line using the miRNeasy Mini Kit protocol (Qiagen, Italy), combining phenol/guanidine-based lysis of samples and silica membrane-based purification of total RNA, including small RNAs. Briefly, C2C12 cells were plated at 5*10^5^ cell density into 100 mm culture dishes (day 0). After 48 h of adhesion, on day 2, DMEM full medium was withdrawn with a differentiation medium. After 48 h, on day 4, differentiated C2C12 were treated with a single dose of HC for 12 h to in vitro mimic a hypercortisolism condition. On day 5, miRNAs were isolated from C2C12 using miRNeasy Mini Kit (code number 217004; Qiagen, Italy). Cell lysis has been induced by QIAzol Lysis reagent whereas the inhibition of RNases and removal of cellular DNA and proteins from the samples were performed during the subsequent phases characterized by the use of different buffers solution and miRNeasy Mini spin column provided by the kit. Total miRNAs bind the spin column’s membrane and contaminants have been efficiently washed away. High-quality miRNAs were then eluted in 20 µl of RNase-free water. 250 ng/µl of miRNAs extracted from cell culture were reversed transcribed using miScript II RT Kit (code number 218161; Qiagen, Italy). The miScript Reverse Transcriptase mix (20ul final volume) contains an internal synthetic RNA control (miRNA reverse transcription control, miRTC) to assess reverse transcription performance during profiling experiments with miScript miRNA PCR Arrays. The reaction tubes were then placed in a thermostat at 37 °C for 1 h, 5 min at 95 °C and then diluted, adding 180 µl of RNAse free water. cDNA derived from the reverse-transcription reaction was used as a template for real-time PCR analysis to evaluate muscle miRNAs (myomiRs) profile expression using mouse miScript miRNA PCR array (Code number 331221 MIMM-106ZC; Qiagen, Italy) and miScript SYBR Green kit (code number 218075; Qiagen, Italy). The PCR mix was prepared by adding 1375 µl 2X QuantiTect SYBR Green PCR Master mix, 275 µl universal primer, 200 µl cDNA and 900 ul H_2_O. Then, 25 µl of PCR mix were distributed to each well to charge 25 ng cDNA per well. The last two wells, containing positive controls, were characterized by the presence of PCR mix and RNAse-free water without the sample. StepOne Plus Real-Time PCR System instrument was used to run the RT-qPCR applying the following protocol: initial activation step at 95 °C (15 min), then samples were subjected to 40 cycles of denaturation at 94 °C (15 s), annealing at 55 °C (30 s) and extension at 70 °C (30 s). Six internal housekeeping genes (SNORD61, SNORD68, SNORD72, SNORD95, SNORD96S and RNU6-6P) were used to normalize miRNAs expression. Results are expressed as the mean of three different experiments. Data analysis and validation were performed using the free online software provided by the manufacturer’s website (Qiagen, Milan, Italy). All miRNAs evaluated by the arrays are listed in Supplementary Table 1.

### miR-133a-3p expression level validation by RT-qPCR in C2C12 cell line

miR-133a-3p expression in C2C12 under treatment with HC alone and in combination with Rel was validated by RT-qPCR. miR-133a-3p were isolated from differentiated C2C12 cell line using the miRNeasy Mini Kit protocol (Qiagen, Italy), as described above. Briefly, C2C12 cells were plated at 3*10^5^ cell density into 60 mm culture dishes (day 0). After 48 h of adhesion, on day 2, DMEM full medium was withdrawn with a differentiation medium. After 48 h, on day 4, differentiated C2C12 were treated with a single dose of Rel (10^−6^ M) for 1 h and HC (1,4*10^−6^ M) for 12 h. On day 5, miRNAs were isolated from C2C12 using miRNeasy Mini Kit (code number 217004; Qiagen, Italy). High-quality miRNAs were then eluted in 30 µl of RNase-free water. 50 ng/µl of miRNAs extracted from cell culture were reversed transcribed using TaqMan^®^ MicroRNA Reverse Transcription Kit (code number 4366596, Applied Biosystem). The MicroRNA Reverse Transcription mix (15 µl final volume) contains RT primers 5X to assess reverse transcription. The reaction tubes were then placed in a thermostat at 16 °C for 30 min, at 42 °C for the other 30 min, and finally at 85 °C for 5 min. cDNAs derived from the reverse-transcription reaction were pre-amplified using TaqMan^®^ PreAmp Master Mix (code number 4488593, Applied Biosystem) and specific pre-formulated TaqMan^®^ MicroRNA Assay 20X (TaqMan^®^ probe and primer set) miR-133a-3p (code number 4427975, assay ID: 002246) and the housekeeping U6 snRNA (code number 4427975, assay ID: 001973). The pre-amplification reaction tubes (final volume 25 µl) were placed in a thermocycler and then incubate using a standard ramp speed and the following settings: enzyme activation at 95 °C (10 min), annealing at 55 °C (2 min), extension at 72 °C (2 min), and 12 cycles of denaturation at 94 °C (15 s), annealing/extension at 60 °C (4 min), and enzyme inactivation at 99.9 °C (10 min). The pre-amplification reaction products were then diluted adding 175 μl of 0.1X TE, pH 8.0 to each tube to reach a final volume of 200 μl. The cDNA pre-amplified was used as a template for real-time PCR analysis to evaluate muscle miR-133a-3p. 2 µl of cDNA were added to 10 µl of PCR mix prepared by adding 0.5 µl of TaqMan^®^ MicroRNA assay 20X, 5 µl PCR master mix (TaqMan^®^ Fast Advanced Master mix, code number 4444964, Applied Biosystem), and 4.5 µl of sterile water.

### Cushing’s syndrome patients

At the Endocrinological and Neurosurgical center of the University of Naples “Federico II”, consecutive patients aged 18–75 years, with clinically evident and biochemically active and proven de novo or persistent CS, were considered eligible and enrolled in the study. The diagnosis of CS was performed with the presence of two of the following criteria, according to the Endocrine Society Guidelines [32]: (1) abnormal (> upper limit of normal (ULN) range) UFC levels in at least two different samples; (2) abnormal (> 1.8 µg/dl) cortisol response to the overnight dexamethasone (Dex) suppression test; (3) abnormal (> 1.8 µg/dl) cortisol response to the 2 mg Dex suppression test. ACTH-independent CS was diagnosed in the presence of low ACTH levels (< 10 pg/ml) whereas ACTH-dependent CS was diagnosed in the presence of hypercortisolism and normal or high ACTH levels [33]. The differential diagnosis between the two forms of ACTH-dependent CS (CD and ectopic CS) was based on the performance of high-dose Dex suppression test and corticotropin-releasing hormone (CRH) stimulation test or desmopressin (DDAVP) stimulation test and on pituitary magnetic resonance imaging (MRI); in case of discordant results at hormonal assessment, absence of pituitary tumour at MRI or pituitary tumour < 6 mm at pituitary MRI, bilateral inferior petrosal sinus sampling (BIPSS) with CRH/DDAVP stimulation was performed. In case of evidence of lack of central: periphery gradient at BIPSS, further imaging assessments, including total-body MRI, CT, 18-FDG-PET, and In111-Octreoscan, were performed to identify the source of ectopic ACTH secretion.

27 patients (21 women and 6 men, aged 44.8 (mean) ± 2.6 (S.E.M.) years) with an active form of CD, 10 patients (8 women and 2 men, aged 55.7 ± 5.5 years) with an active form of CS, and 24 healthy subjects (17 women and 8 men, aged 44.5 ± 2.1 years), recruited from the staff and the faculty of the University of Naples “Federico II” as a control group, were enrolled in the study. Patients and controls were age- and sex-matched as reported in Table 1. CD and CS patients provided the routine hormonal status measurements: morning serum ACTH and cortisol were analyzed by immunochemiluminescence assay (CLIA) using Siemens Immulite 2000^®^ and Siemens Advia Centaur^®^, respectively. The 24 h UFC was measured by CLIA using Siemens Advia Centaur^®^ after extraction with methylene chloride with different reference ranges, therefore, the values of the ULN were considered.

**Table 1:** Clinical characteristics of CD and CS patients and healthy subjects

	Healthy Subjects	Cushing’s Disease (CD)	Cushing's Syndrome (CS)	*P*
*N*	24	27	10	
Sex F(%):M(%)	17(70.8%):8(33.3%)	21(77.7%):6(22.2%)	8(80%):2(20%)	0.73
Age mean ± SEM	44.5 ± 2.1	44.8 ± 2.6	55.7 ± 5.5	0.12
BMI mean ± SEM	24.1 ± 0.6	32.3 ± 2.1****	27.8 ± 1.0*	*0.02; ****0.0008
ACTH pg/ml (09:00 h) mean ± SEM	N.A	162.1 ± 52.7^††††^	17 ± 9.0	0.0001
Cortisolo ng/ml (09:00 h) mean ± SEM	N.A	165 ± 18.3	226.9 ± 26.8	0.27
24 h UFC (ULN mean and range)	N.A	2.88 (0.41–11.6)	1.53 (0.35–3.1)	0.11

*p = 0.02 and ****p = 0.0008 vs healthy subjects. ††††*p* < 0.0001 CD vs CS

### Samples collection, serum miRNAs isolation and quantification

All subjects had fasting blood samples collected in the morning into EDTA tubes. Samples were centrifuged at 4 °C and 2000 rpm for 5 min within two hours of collection to preserve miRNA integrity, then aliquoted and stored at − 80 °C until further analysis. Circulating miRNAs were isolated from 200ul of human plasma using miRNeasy Serum/Plasma Advanced Kit (code number 217204; Qiagen, Italy). The miRNeasy Serum/Plasma Advanced Kit efficiently isolates total RNA, including miRNAs, in and outside vesicles providing a phenol-free protocol and easy-to-automate MinElute spin column technology. Spike-in control cel-miR-39-3p, at a working solution equal to 1.6*10^8^ copies, was added to plasma during the lysis step of miRNA extraction. 50 ng/ul of recovered miRNAs were used for cDNA synthesis using the miScript system (code number 218161; Qiagen, Italy), followed by cDNA preamplification. 5ul of cDNA was used for preamplification using the miSript PreAMP PCR kit (code number 331452; Qiagen Italy) to have a final reaction volume of 25ul. PCR preamplification reaction run was designed by 1) activation step (15 min at 95 °C); 2) 12 cycles of denaturation (30 s at 94 °C) and annealing stage (3 min at 60 °C). The final amplification product has been diluted 1:5 adding 100ul of RNAse free water. cDNA derived from the preamplification reaction was used as a template for RT-qPCR analysis to evaluate circulating human myomiRs miR-133a-3p (code number 218300 Ms00031423, Qiagen, Italy), miR-122-5p (code number 218300 Ms00003416, Qiagen, Italy), miR-200b-3p (code number 218300 Ms00009016; Qiagen, Italy) expression using the miScript SYBR Green kit (code number 218075; Qiagen, Italy). RT-qPCR reaction was performed according to the following protocol: 1) activation step (15 min at 95 °C); 2) denaturation step (15 s at 94 °C), 3) annealing step (30 s at 55 °C), 4) extension step (30 s at 70 °C). Reactions were run in duplicate on a StepOne Plus RT-qPCR machine (Applied Biosystems Foster City, CA, USA). The relative expression levels of each transcript analyzed in each sample were normalized using the spike-in control cel-miR-39-3p.

### Statistical analysis

The analysis of the PCR Array results was performed using Qiagen software. The Volcano plot analysis, which combines the measure of statistical significance from the statistical test with the magnitude of the fold change, was performed. The analysis of the difference between the study’s groups was performed using student’s t test and Mann–Whitney (GraphPad Prism version 6 software). ROC analysis was performed to assess the potential diagnostic accuracy of circulating miRNAs expression levels, the AUC, a measure to distinguish between two diagnostic groups, and the cut-off values. The correlation of miRNAs expression levels with 24 h UFC, expressed as ULN, was tested with Pearson’s correlation and linear regression analysis to identify predictors of CS. We considered *p* to be significant at < 0.05 with a 95% confidence interval (CI).

## Results

### HC treatment induces atrophic signals in C2C12

**Fig. 1:**
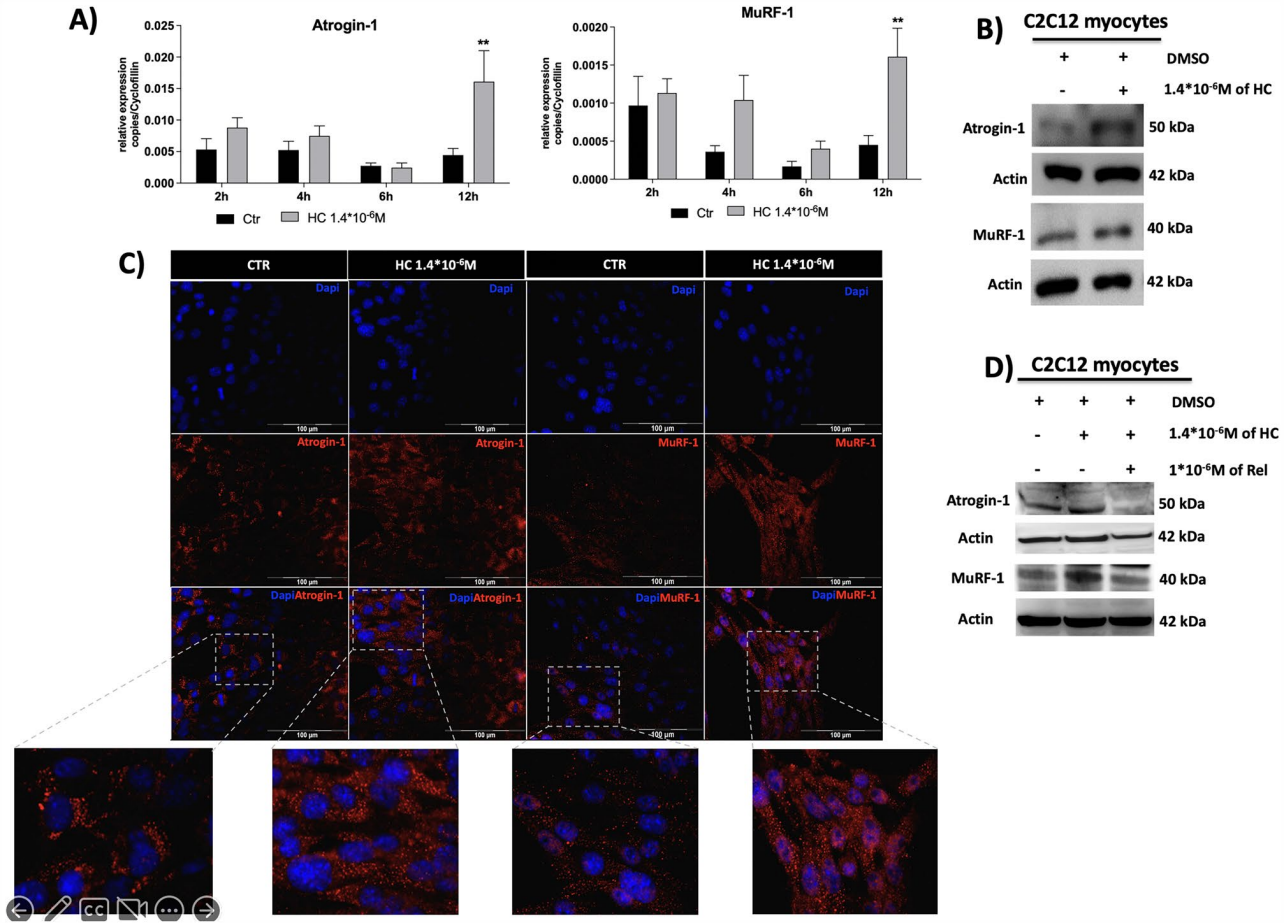
Atrogin-1 and MuRF-1 gene expression after 2, 4, 6 and 12 h of HC treatment (**A**) and protein expression after 12 h of HC treatment (**B** and **C**) and after 1 h of Rel before the 12 h of HC treatment (**D**) in C2C12 cells differentiated in myocytes. **p < 0.01 vs control. *HC* hydrocortisone, *Rel* relacorilant

To investigate the HC-mediated atrophic signalling the mRNA and protein expression of the atrogenes, Atrogin-1 and MuRF-1, were assessed in C2C12 myocytes treated with HC at different time points. Figure 1A shows that HC 1.4*10^–6^ M significantly induced Atrogin-1 and MuRF-1 mRNA (*p* < 0.01) expression, evaluated by RT-qPCR, after 12 h of treatment compared to controls; as well as Fig. 1B, C show that HC significantly induced Atrogin-1 and MuRF-1 protein expression, evaluated by both western blot (WB) and immunofluorescence (IF), after 12 h of treatment compared to controls. Moreover, Fig. 1D shows that the overexpression of Atrogin-1 and MuRF-1 proteins was due to the specific HC effect through the GR binding, indeed the pre-treatment of GR antagonist Rel prevented HC effect.

### HC treatment induces upregulation of myomiRs in C2C12 myocytes

Established that HC induced atrophic signals, as demonstrated by the increase of mRNA and protein expression of Atrogin-1 and MuRF-1 genes, in in vitro mouse C2C12 myocytes, the analysis of intracellular myomiRs has been performed to investigate the HC effect of myomiRs production by muscle cells.

**Fig. 2:**
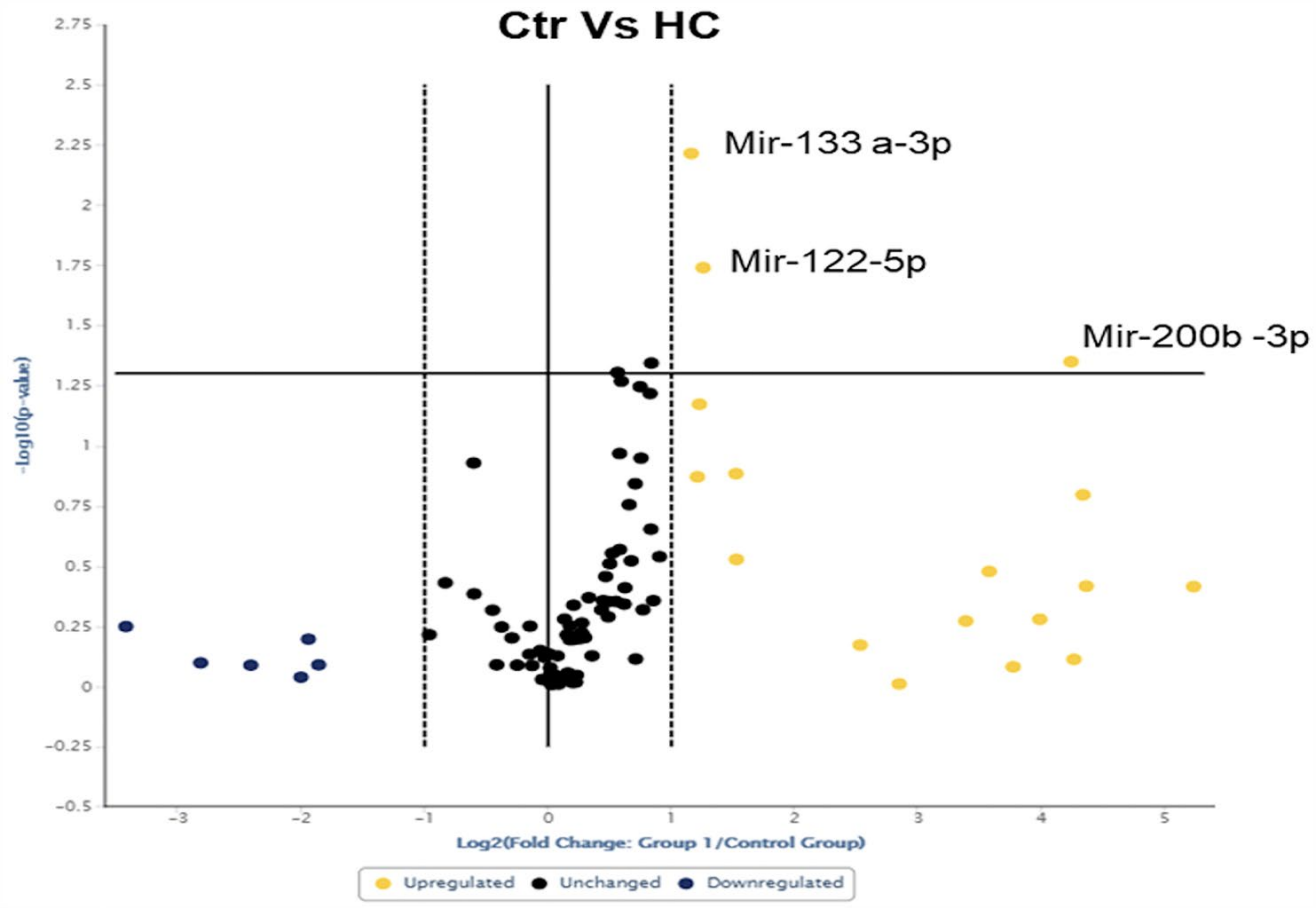
**A** Volcano plot representation indicates the statistical significance of myomiRs expression changes comparing differentiated C2C12 cells exposed to HC for 12 h and untreated cells. The yellow dots in the upper-right quadrant represent the myomiRs significantly upregulated. miR-133a-3p, miR-122-5p and miR-200b-3p resulted significantly upregulated

Figure 2 shows the Volcano plot analysis. The results revealed that HC treatment at 1.4*10^–6^ M for 12 h in C2C12 myocytes significantly induced the overexpression of three myomiRs: miR-133a-3p (*p* = 0.0061), miR-122-5p (*p* = 0.0188) and miR-200b-3p (*p* = 0.0447).

### Routine hormonal measurements and circulating myomiRs in CS patients and controls

**Fig. 3:**
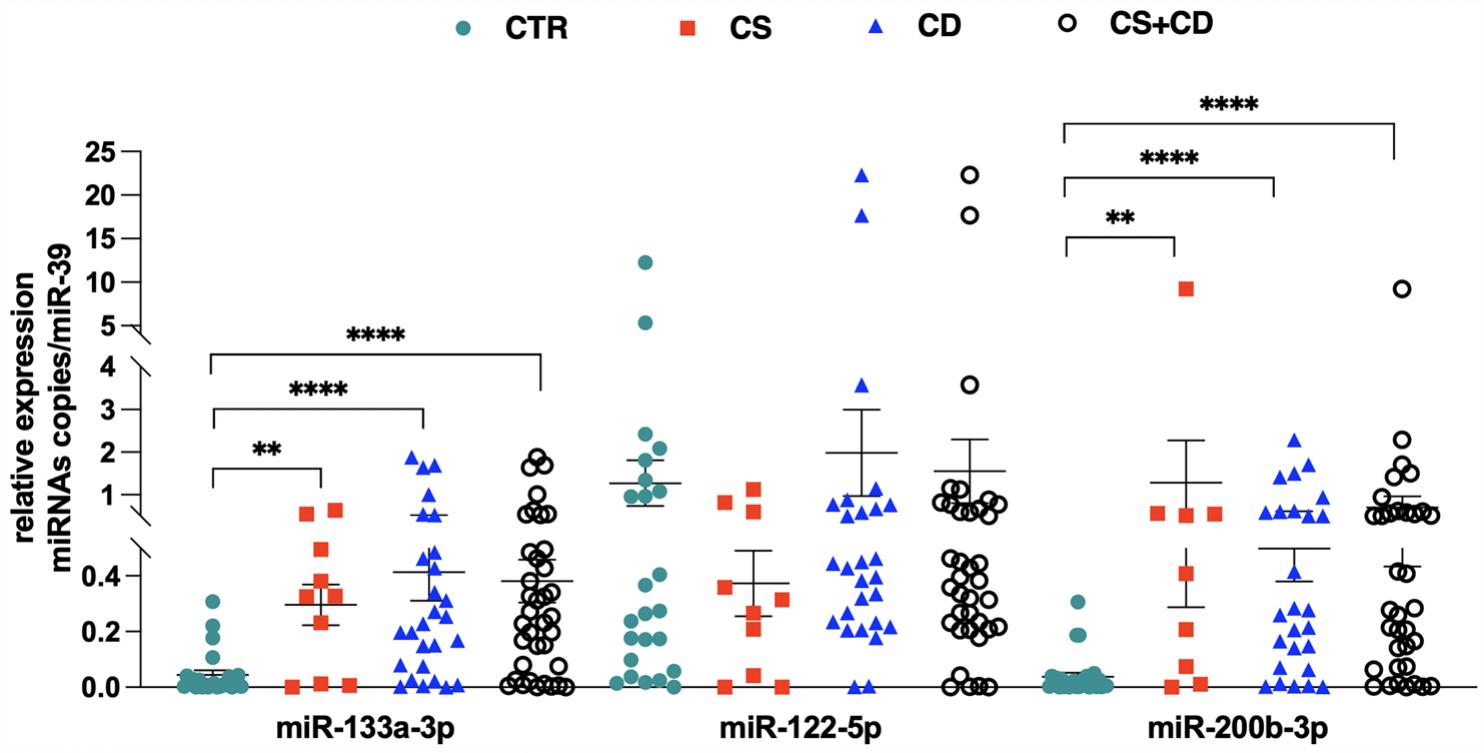
Circulating miR-133a-3p, miR-122-5p and miR-200b-3p levels in CS (also stratified in CS and CD groups) vs controls. miR-133a-3p: **p = 0.007; ****p < 0.0001. miR-200b-3p: **p = 0.002; ****p < 0.0001. CD: Cushing’s disease. *CS* Cushing’s syndrome

**Fig. 4:**
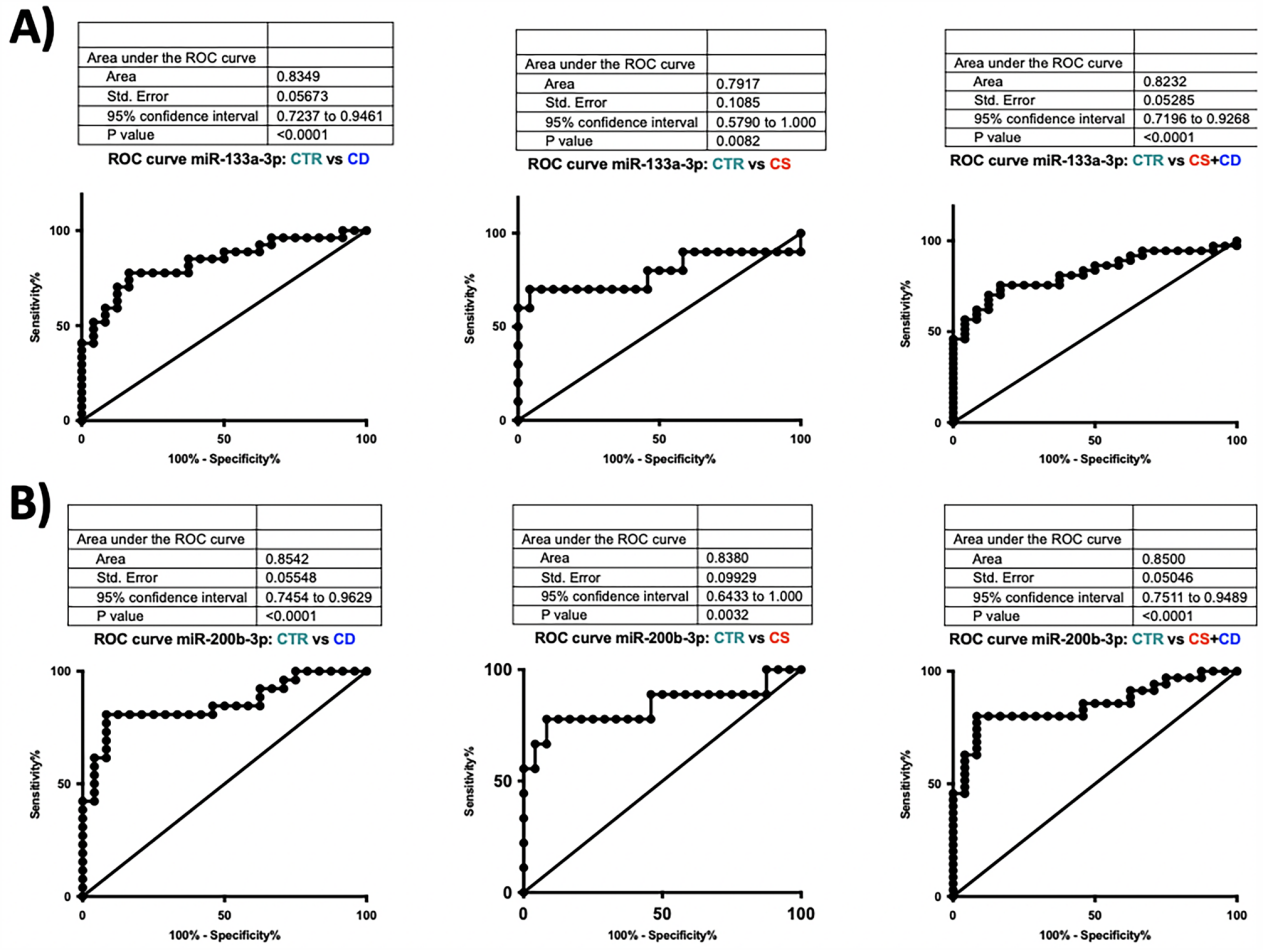
Evaluation of the diagnostic applicability by receiver operating characteristic (ROC) curves of the two myomiRs miR-133a-3p (**A**) and miR-200b-3p (**B**). ROC curves were plotted for CS (CS + CD grouped together) vs controls, CD vs controls and CS vs controls

A total of 61 participants were enrolled in the study: 37 patients with CS, including 27 patients with pituitary ACTH-dependent CS (CD) and 10 patients with ACTH-independent CS (adrenal CS), and 24 healthy subjects as control group. Patients’ clinical features are reported in Table 1. A significant increase in the circulating levels of miR-133a-3p (*p* < 0.0001) and miR-200b-3p (*p* < 0.0001) was found in the totality of CS patients compared to controls. Moreover, when the patients were stratified into CD and adrenal CS groups the circulating levels of miR-133a-3p and miR-200b-3p were significantly higher in both CD (*p* < 0.0001, *p* < 0.0001) and adrenal CS (*p* = 0.007, *p* = 0.002) patient groups, respectively, compared to controls. No significant difference in miR-122-5p circulating levels was found between either the totality of CS patients or the stratified CD and adrenal CS groups compared to controls. Figure 3 shows scatter dot plots for miR-133a-3p, miR-122-5p and miR-200b-3p in the totality of CS patients compared to controls and in patients stratified in CD and adrenal CS groups. To investigate the potential diagnostic value of the two selected myomiRs (miR-133a-3p and miR-200b-3p), receiver operating characteristics (ROC) curves were performed, and the area under the curve (AUC) values were calculated in patients with CD and adrenal CS and control groups. When samples were pooled together as CD *plus* adrenal CS group, the cut-off value for miR-133a-3p was 0.043 with a specificity of 83.33% and a sensitivity of 75.68% and the AUC was 0.823 (95% CI 0.719–0.926, *p* < 0.0001 vs CD *plus* adrenal CS), whereas the AUC was 0.835 in CD group (95% CI 0.724–0.946, *p* < 0.0001 vs adrenal CD) and 0.792 in adrenal CS group (95% CI 0.579–1.000, *p* = 0.008 vs adrenal CS). Similarly, when samples were pooled together as CD *plus* adrenal CS the cut-off value for miR-200b-3p was 0.049 with a specificity of 91.67% and a sensitivity of 80.00% and the AUC was 0.850 (95% CI 0.751–0.949, *p* < 0.0001 vs CD *plus* adrenal CS), whereas the AUC was 0.854 in CD group (95% CI 0.745–0.963, *p* =  < 0.0001 vs CD) and 0.838 in adrenal CS group (95% CI 0.643–1.000, *p* = 0.0032 vs adrenal CS). However, ROC curves demonstrated that neither miR-133a-3p nor miR-200b-3p represent potential biomarkers to discriminate between CD and adrenal CS. Figure 4 shows ROC curves of miR-133a-3p and miR-200b-3p in the totality of CS patients and in patients stratified in CD and adrenal CS groups versus the control group.

### Correlation study

**Fig. 5:**
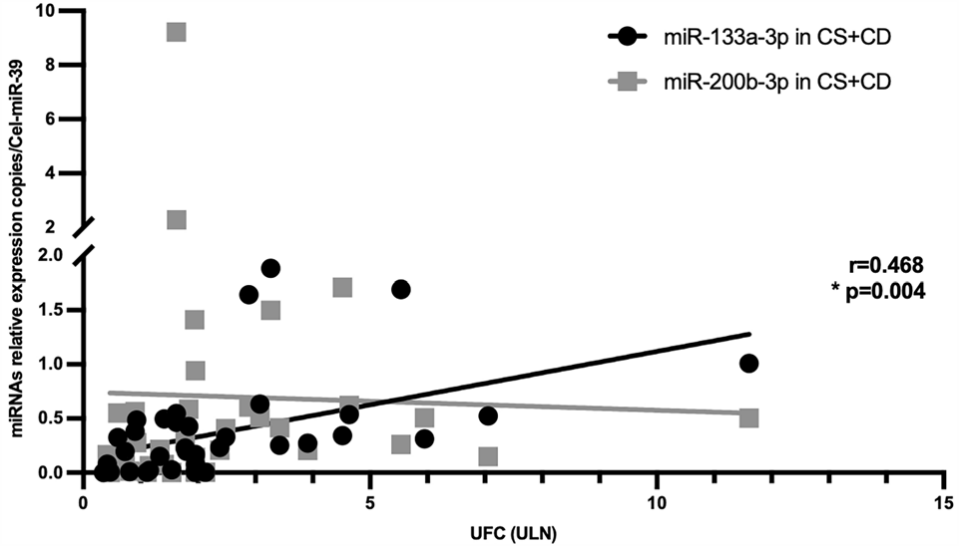
Correlation and linear regression of miR-133a-3p and miR-200b-3p serum circulating levels and 24 h UFC (ULN). UFC: urinary free cortisol. ULN: upper limit of normal

The linear regression analysis with Pearson correlation (*r* = 0.468, *p* = 0.004) revealed that miR-133a-3p circulating levels were positively correlated with 24 h UFC levels expressed as upper limit of normal (ULN) in the totality of CS patients. This significant association persisted even after correction for the body mass index (BMI) (*r* = 0.487, *p* = 0.012). No significant correlation was found between miR-200b-3p circulating levels and 24 h UFC levels expressed as ULN in the totality of CS patients. Figure 5 depicts a scatter plot with linear regression with fitted regression lines for miR-133a-3p and miR-200b-3p. Pearson’s correlation coefficient (r) and significance (p) has been reported for miR-133a-3p.

### Bioinformatic prediction of miR-133a-3p target

**Fig. 6:**
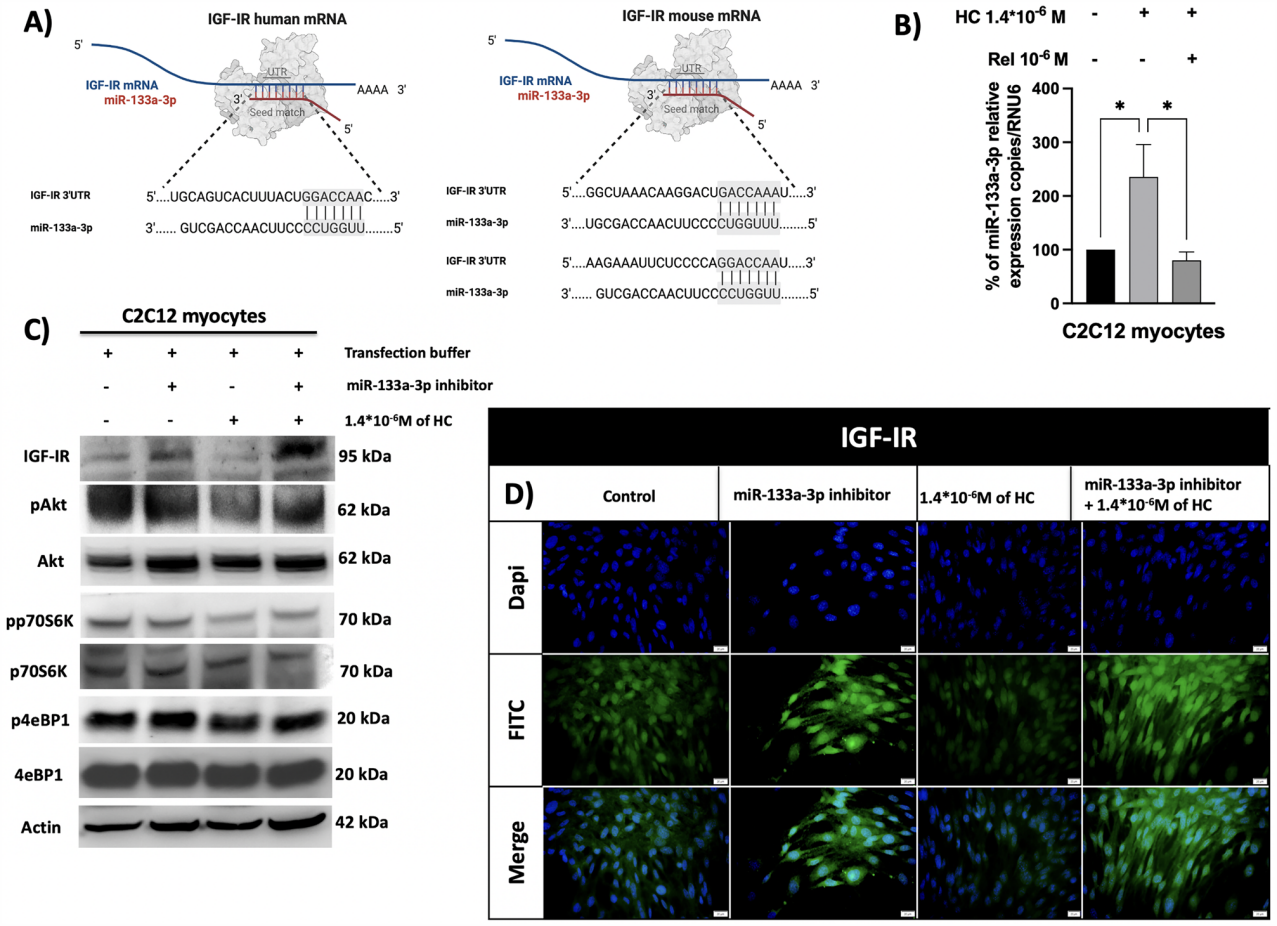
**A** miR-133a-3p seed match in 3’UTR of IGF-IR mRNA in humans and mice. Created with BioRender.com, **B** miR-133a-3p expression in C2C12 treated with HC alone and in combination with Rel expressed as a percentage compared to control. HC increased the levels of miR-133a-3p by about 235% compared to control (*p = 0.035); the use of Rel blocked the effect of HC (*p = 0.023, HC vs HC + Rel). **C** IGF-IR and PI3K/AKT/mTOR components protein expression in C2C12 transfected with miR-133a-3p inhibitor and treated with HC. Inhibition of miR-133a-3p induced IGF-IR protein up-regulation in both C2C12 cells. IGF-IR protein, instead, was down-regulated in presence of HC and transfection buffer. Conversely, IGF-IR protein was up-regulated in presence of both miR-133a-3p inhibitor and HC. **D** The blocking of miR-133a-3p induced a strong IGF-IR cytoplasmic and nuclear staining, compared to the control which showed a weaker cytoplasmic localization and poorer nuclear localization. HC treatment reduced IGF-IR protein staining compared to the control. In the presence of miR-133a-3p inhibitor, HC could no longer able to mediate its inhibitor effect on IGF-IR protein expression, restoring IGF-IR cytoplasmic and nuclear protein expression localization. *HC* hydrocortisone. *Rel* relacorilant

Having proven that miR-133a-3p circulating levels are significantly high and positively correlated with 24 h UFC levels in CS patients, the potential detrimental role of miR-133a-3p on muscle mass was investigated. To identify potential target genes of miR-133a-3p, the bioinformatic TargetScan 7.2 software was used. Among all targets, in humans, IGFI receptor (IGF-IR) results to be a target gene of miR-133a-3p with a seed region in 3’UTR sequence, as depicted in Fig. 6A. In mouse, TargetScan 7.2 software does not predict the miRNA seed match in the same region, but a careful study of the 3’ UTR sequence using the bioinformatic bank Ensembl highlighted the presence of a possible miR-133a-3p seed region in mouse IGF-IR mRNA, as depicted in Fig. 6A. The binding of miR-133a-3p to IGF-IR might reduce its protein expression acting post-transcriptionally, therefore, reducing the anabolic action of IGFI through PI3K/Akt/mTOR pathway on muscle mass and, consequently, muscle performance.

### miR-133a-3p contributes to HC-mediated muscle atrophic signals in C2C12 myocytes

Demonstrated that, among the three upregulated miRNAs by HC in C2C12, miR-133a-3p was significantly upregulated in the serum of CS patients, the modulation of miR-133a-3p expression in C2C12 was validated by RT-qPCR in presence of HC alone and in combination with Rel. Figure 6B shows the significant (*p* = 0.035) effect of HC in inducing miR-133a-3p upregulation compared to the control. Moreover, this effect appeared to be specifically HC-mediated, indeed completely abolished when a selective GR modulator, such as Rel, was used.

To evaluate whether the overexpression of miR-133a-3p induced by HC could lead to downregulation of IGF-IR expression in myocytes, causing a decreased PI3K/Akt/mTOR signalling and consequent muscle weakness and further atrophy, protein of IGF-IR and downstream PI3K/Akt/mTOR components were evaluated by IF and WB analyses, both in the condition of HC-mediated miR-133a-3p overexpression and miR-133a-3p knocking-down in myocytes. The results of WB analysis confirmed that blocking miR-133a-3p, performed by transfection with miR-133a-3p inhibitor, prompted overexpression of IGF-IR protein, accompanied by the sustained activation of Akt, p70S6K and 4eBP1 proteins in C2C12 myocytes. Conversely, according to the induction of atrophic signals, HC treatment reduced IGF-IR, pAkt, pp70S6K and p4eBP1 protein expression. Interestingly, in presence of miR-133a-3p inhibitor, HC was no longer able to downregulate IGF-IR, pAkt, pp70S6K and p4eBP1 suggesting that HC indirectly mediated muscle anti-anabolic signals downregulating IGF-IR protein expression through the overexpression of miR-133a-3p and, consequently, reducing the activation of the anabolic PI3K/Akt/mTOR pathway. Figure 6C shows the protein expression of the different components evaluated by WB in C2C12 myocytes subjected to different treatments: miR-133a-3p inhibition, HC treatment and this latter in presence of miR-133a-3p inhibitor. IGF-IR protein expression results were confirmed by IF analysis. Interestingly, blocking of miR-133a-3p with inhibitor transfection induced a more widespread IGF-IR cytoplasmic and nuclear staining, compared to control which showed a weaker cytoplasmic localization and a poorer nuclear localization. Conversely, HC treatment drastically reduced both IGF-IR protein localization compared to the control. Interestingly, in presence of miR-133a-3p inhibitor, HC was no longer able to mediate its inhibitor effect on IGF-IR protein expression, as demonstrated by the restored IGF-IR cytoplasmic and nuclear protein expression localization. Figure 6D shows the IGF-IR protein expression evaluated by IF in C2C12 myocytes subjected to different treatments: miR-133a-3p inhibition, HC treatment and this latter in the presence of miR-133a-3p inhibitor.

## Discussion

CS patients are characterized by multiple features that in part include typical pathognomonic clinical signs and symptoms, generally less apparent in men than in women, and in part comprise common features diffused in a large prevalence in the general population, still making the diagnosis a challenging task [34, 35]. According to the clinical practice guidelines [32, 36], the diagnostic algorithm of CS first aims at identifying the CS, by confirming the hypercortisolism, and secondly at recognizing the CS aetiology. Diagnosis of CS is established on the results of two or more concordant diagnostic tests entailing the cortisol secretory status together with different robust imaging procedures. Biochemical diagnosis is based on the results of three approaches most often used to evaluate hypercortisolism: (1) assessing cortisol excretion in a 24 h period by collecting two or three urine samples, (2) documenting the loss of feedback inhibition of cortisol on the hypothalamus–pituitary–adrenal axis with Dex suppression testing, and 3) documenting the loss of normal rhythm in cortisol secretion assessing the late-night salivary cortisol levels by collecting two or three salivary samplings [36]. Anyhow, there is neither a single preferred diagnostic test nor a consensus on how to decide whether and when to test [36]. Moreover, the incomplete accuracy of these biochemical diagnostic procedures, with specificity lower than sensitivity, makes the diagnosis difficult, particularly in earlier and mild cases. Therefore, new biomarkers and diagnostic strategies could help and facilitate an accurate diagnosis, even in milder cases of CS that often present diagnostic challenges due to overlapping features with various other pathological conditions.

In this scenario, the evaluation of circulating levels of specific miRNAs between CS and the general population could pave the way for the potential usefulness of miRNA-expression profile, as new potential non-invasive biomarkers and eventually represent a new easy diagnostic tool for the identification of CS.

Recently, only three studies have investigated the circulating miRNAs expression in CS with the aim to use them as biomarkers [26, 27, 37]. However, this is the first study, focusing on the analysis of a specific miRNA subset, the myomiRs, considering that GC-induced myopathy can occur in an acute or chronic form in mild and severe cases, starting from in vitro findings obtained in a condition mimicking hypercortisolism. This preliminary in vitro evaluation of myomiR subset performed in a mouse cell model is to be considered highly representative of the human condition as the myomiRs evaluated, especially that found to be significantly overexpressed (miR-133a-3p, miR-200b-3p, miR-122-5p) are highly conserved among vertebrate species [22, 38, 39].

The results of the current study showed relevantly different serum circulating levels of two myomiRs (miR-133a-3p and miR-200b-3p) with a significantly higher expression of their circulating levels in patients with CS compared with healthy subjects. Moreover, using receiver operating characteristic (ROC) curve analysis, both miR-133a-3p and miR-200b-3p turned out to be useful diagnostic biomarkers to discriminate CS from healthy subjects considering their good diagnostic performance. Finally and more interestingly, these are the first results, to the best of our knowledge, demonstrating that miR-133a-3p could represent a useful circulating biomarker to indicate the disease severity, resulting positively correlated with 24 h UFC levels, although there is no general scientific consensus on the use of the UFC levels as biomarkers of CS severity [40, 41].

The results of the current study are in line with other findings in which miR-133a-3p has been previously reported to be overexpressed in several pathological diseases, such as myotonic dystrophy, spinal muscular atrophy and amyotrophic lateral sclerosis, characterized by muscle weakness and progressive muscle wasting [23–25].

In apparent contradiction, neither miR-133a-3p nor miR-200b-3p appeared to be reported significantly dysregulated when the miRNA profiling was evaluated by Vetrivel et al*.* in a cohort of 15 CS patients, each before and after curative surgery [27], and by Hara et al*.* in a cohort of 5 patients with primary bilateral macronodular adrenocortical hyperplasia, compared with patients harbouring adenomas with and without cortisol excess [37], by next-generation sequencing (NGS). Belaya et al. investigated several circulating miRNAs, including miR-133a-5p but not miR-133a-3p [26]. The discrepancy in the outcome of the results between the studies could be due to different variables: (1) variation in the amount of starting material, samples’ number and collection, miRNA extraction, reverse transcription, and amplification efficiency, as well as technology might introduce bias, and consequently, could lead to misleading conclusions and impaired comparison between studies. In this specific case, the discrepancies between the results of the current study and the results of Vetrivel and Hara could be addressed by the different technologies used: the NGS technology, which allows the detection of a large set of miRNAs and the discovery of novel miRNAs, and the RT-qPCR, performed in the current study, that due to its high sensitivity and specificity, is the current gold standard method also used to verify data obtained by microarrays or NGS approaches.

Moreover, it is noteworthy that variations in the circulating miRNA profile have been described in several chronic metabolic conditions, including obesity [42]. As chronic hypercortisolism determines a redistribution of body fat deposition leading to increased abdominal adiposity, with the related metabolic consequences, the circulating miRNA profile in CS patients could be strongly affected by obesity or overweight condition. Nevertheless, in the current study the regression analysis performed to assess the linear correlation between miR-133a-3p and 24 h UFC levels, was performed correcting the data for the BMI, demonstrating an independent role of weight status. The limitations of the *ex-vivo* section of the current study could be represented by: (1) the lack of a power sample analysis due to the pilot nature of the current study on a rare disease such as CS; (2) the use of a control group missing general comorbidities that could be even secondary to hypercortisolism, for instance, diabetes or hypertension and that could influence the circulating miRNA levels and (3) the lack of the waist circumference records for the patients and control group which limited the correction of the analysis of circulating miRNA levels only for BMI and not for the abdominal adiposity, a hallmark of CS.

However, the current study focused on a small subset of myomiRs, whose levels were specifically altered and secreted by muscle tissue in the condition of cortisol excess, as assessed by the in vitro data. A catabolic condition such as GCs excess promotes muscle atrophy that results from decreased protein synthesis, sustained by the different pathways, including IGFI/PI3K/Akt/mTOR pathway, and increased protein degradation, mainly supported by ubiquitin–proteasome and autophagy-lysosome systems [11]. The potential mechanistic autocrine, paracrine and/or endocrine role of the high circulating miR-133a-3p and miR-200b-3p levels on different peripheral tissues in CS patients has not been previously reported. Interestingly, in muscles, the role of miR-133a-3p during myogenesis has been demonstrated in C2C12 cells in which miR-133a-3p modulates IGFI/PI3K/Akt/mTOR signalling through repression of IGF-IR, negatively modulating the anabolic IGFI pathway [43]. GRs have previously been demonstrated to be expressed in C2C12 myocytes and activated by Dex [44]. GR expression has been proven to be essential to elicit Dex-mediated atrophic signals promoting the upregulation of Atrogin-1 and MuRF-1 protein expression (18) and the expression of miR-322 with consequent negative deregulation of IGF-IR and insulin receptors in C2C12 myotubes [44]. The in vitro results of the current study demonstrate that HC, through the binding of GRs, induces muscle atrophy both by directly activating Atrogin-1 and MuRF-1, inducing protein degradation and by indirectly prompting the IGF-IR protein downregulation, therefore decreasing protein synthesis via IGFI/PI3K/Akt/mTOR pathway. Interestingly, the regulation of IGF-IR protein expression, and consequently the downstream PI3K/Akt/mTOR pathway, requires the activation of the myomiR miR-133a-3p.

Moreover, remarkably, an endocrine role of circulating miR-133a-3p has also been identified in the context of bipolar disorder (BD), a neuropsychiatric disorder observed in 30% of active CS patients [4], and in schizophrenia [45], whose typical psychotic state has also been detected in case of CS [46]. Finally, among the potential targets of miR-133a-3p, there is the co-chaperone protein FK506 binding protein 5 (FKBP5), notably involved in the reduction of binding affinity between GCs and GR, by altering the folding of GR and, thus, suppressing GR nuclear translocation and the consequential action as transcription factor [47]. Consequently, the epigenetic role of miR-133a-3p on the FKBP5 gene might reduce the expression of FKBP5 protein leading to a potential increase in the availability of GR in binding GCs, in an amplified binding and nuclear translocation of GCs-GR complex to glucocorticoid-response elements on DNA, with a significant impact on a broad spectrum of physiological and pathophysiological processes, including the lack of a negative feedback efficiency. Therefore, it could be speculated that in CS patients, the high circulating miR-133a-3p levels might (1) induce an autocrine catabolic action on skeletal muscles inducing atrophic signalling; (2) induce an endocrine role on brain-specific pathophysiological mechanisms of illness, mainly BD and schizophrenia; and (3) sustain the prolonged GCs effects negatively regulating the post-transcriptional activity of FKBP5.

On the other hand, the role of miR-200b-3p in cancer progression and drug resistance is widely reported, whereas no data on potential functions, neither on muscle differentiation and regeneration signalling, nor on neuropsychiatric disorders underlying mechanisms or on the regulation of GCs effects are presently reported in the literature.

## Conclusions

**Fig. 7:**
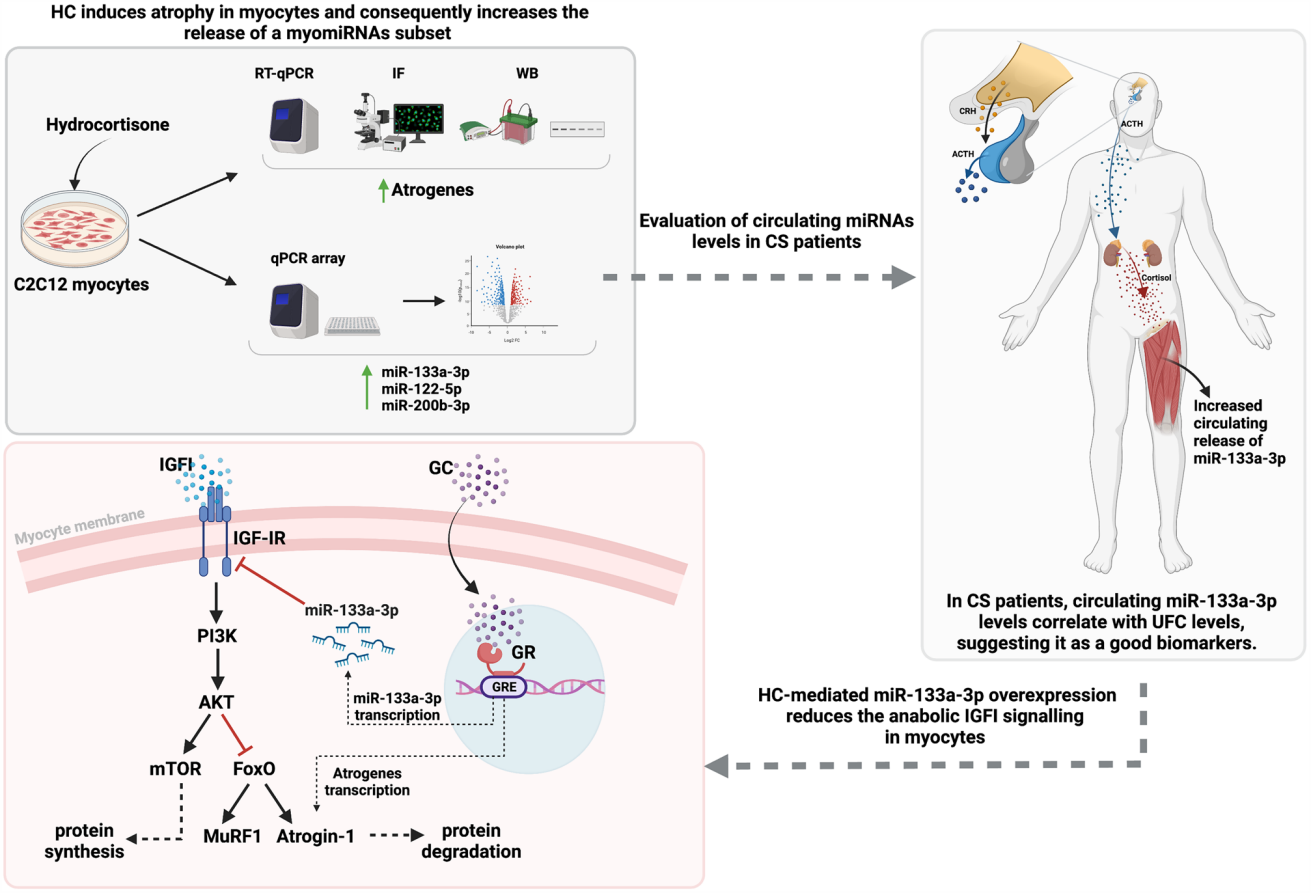
Summary of HC effect on myomiRs expression and function in C2C12 muscle cells and on circulating levels of myomiRs in CS patients

In conclusion, as summarized in Fig. 7, given the remarkable potential diagnostic properties, including easy access, high stability in body fluids, resistance to storage handling, tissue specificity and significant sensitivity, the findings of the current study suggest that miR-133a-3p and miR-200b-3p might be new promising biomarkers to differentiate between CS patients and healthy subjects and that, particularly, miR-133a-3p is a predictive biomarker of hypercortisolism. Moreover, the results of the current study demonstrate that the HC-induced miR-133a-3p elevated levels mediate the muscle atrophic signals by reducing the anabolic effects triggered by the IGF-IR signalling in the muscle cell model, C2C12, thus suggesting the same role of miR-133a-3p in CS patients.

### Supplementary Information

Below is the link to the electronic supplementary material.

Supplementary file1 (XLS 31 KB)

## Data Availability

The datasets used and/or analysed during the current study are available from the corresponding author upon reasonable request.

## Abbreviations

ACTH: Adrenocorticotropic hormone
AUC: Area under the curve
BIPSS: Bilateral inferior petrosal sinus sampling
BMI: Body mass index
CD: Cushing’s disease
CI: Confidence interval
CLIA: Immunochemiluminescence assay
CRH: Corticotropin-releasing hormone
CS: Cushing’s syndrome
Dex: Dexamethasone
FKBP5: FK506 binding protein 5
FoxO: Forkhead box protein O proteins
GCs: Glucocorticoids
GR: Glucocorticoid receptor
HC: Hydrocortisone
IF: Immunofluorescence
IGF-IR: Insulin-like growth factor I receptor
IGFI: Insulin-like growth factor I
MAFbx/Atrogin-1: Muscle atrophy F-box
MRI: Magnetic resonance imaging
MuRF1: E3 ubiquitin ligases muscle RING finger 1
myomiRs: Muscle-specific microRNAs
NGS: Next-generation sequencing
Rel: Relacorilant
ROC: Receiver operating characteristic curves
RT-qPCR: Quantitative reverse transcriptase–polymerase chain reaction
UFC: Urinary free cortisol
ULN: Upper limit of normal
UPS: Ubiquitin–proteasome system
WB: Western blot
